# Recent progress on the functionalization of white phosphorus in China

**DOI:** 10.1093/nsr/nwae162

**Published:** 2024-05-02

**Authors:** Xinlei Huangfu, Zhongzhen Wang, Yu Chen, Junnian Wei, Wei Liu, Wen-Xiong Zhang

**Affiliations:** Beijing National Laboratory for Molecular Sciences (BNLMS), State Key Laboratory of Rare-Earth Materials Chemistry and Applications & Key Laboratory of Bioorganic Chemistry and Molecular Engineering of Ministry of Education, College of Chemistry and Molecular Engineering, Peking University, Beijing 100871, China; Beijing National Laboratory for Molecular Sciences (BNLMS), State Key Laboratory of Rare-Earth Materials Chemistry and Applications & Key Laboratory of Bioorganic Chemistry and Molecular Engineering of Ministry of Education, College of Chemistry and Molecular Engineering, Peking University, Beijing 100871, China; Beijing National Laboratory for Molecular Sciences (BNLMS), State Key Laboratory of Rare-Earth Materials Chemistry and Applications & Key Laboratory of Bioorganic Chemistry and Molecular Engineering of Ministry of Education, College of Chemistry and Molecular Engineering, Peking University, Beijing 100871, China; Beijing National Laboratory for Molecular Sciences (BNLMS), State Key Laboratory of Rare-Earth Materials Chemistry and Applications & Key Laboratory of Bioorganic Chemistry and Molecular Engineering of Ministry of Education, College of Chemistry and Molecular Engineering, Peking University, Beijing 100871, China; Beijing National Laboratory for Molecular Sciences (BNLMS), State Key Laboratory of Rare-Earth Materials Chemistry and Applications & Key Laboratory of Bioorganic Chemistry and Molecular Engineering of Ministry of Education, College of Chemistry and Molecular Engineering, Peking University, Beijing 100871, China; Beijing National Laboratory for Molecular Sciences (BNLMS), State Key Laboratory of Rare-Earth Materials Chemistry and Applications & Key Laboratory of Bioorganic Chemistry and Molecular Engineering of Ministry of Education, College of Chemistry and Molecular Engineering, Peking University, Beijing 100871, China

**Keywords:** white phosphorus, organophosphorus compound, activation, selectivity, efficiency

## Abstract

Direct synthesis of organophosphorus compounds from white phosphorus represents a significant but challenging subject, especially in the context of ongoing efforts to comprehensively improve the phosphorus-derived chemical industry driven by sustainability and safety concerns. China is the world's largest producer of white phosphorus, creating a significant demand for the green transformation of this crucial feedstock. This review provides an overview of advancements in white phosphorus activation by Chinese research teams, focusing on the direct construction of P‒C/N/O/S/M bonds from white phosphorus. Additionally, we offer some insights into prospective directions for the activation and transformation of white phosphorus in the future. This review paper aims to attract more researchers to engage in this area, stimulating follow-up exploration and fostering enduring advances.

## Teaser

This review outlines the advances of white phosphorus chemistry in China and offers insights into future developments, serving as a window to comprehend the contributions from Chinese researchers in this field.

## INTRODUCTION

**Figure 1.**
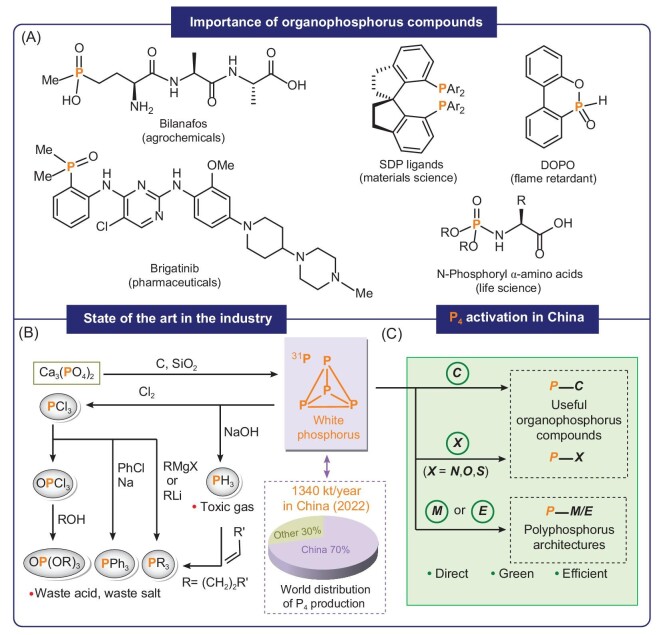
The importance of OPCs and comparison of different routes to commercial OPCs.

Organophosphorus compounds (OPCs) are ubiquitous in daily life, and have numerous applications in pharmaceuticals, food additives, pesticides, flame retardants, electrolytes and detergents [1–5]. They also contribute significantly to fundamental research, particularly in synthetic chemistry [6–8], materials science [9] and abiogenesis [10], propelling the evolution of chemistry and life science (Fig. 1A). The present-day synthesis of OPCs predominately relies on white phosphorus (P_4_), discovered by German alchemist Hennig Brand in 1669 [11]. And now, as the main feedstock chemical, P_4_ is obtained annually in excess of 1 million tons through the reduction of phosphate rock [12]. In conventional processes, (oxy)chlorination of P_4_ results in the formation of PCl_3_, PCl_5_ and OPCl_3_, each posing a considerable environmental risk due to their volatile and hazardous liquid nature. Subsequent stages entail the generation of HCl or salt, culminating in the production of a diverse array of value-added fine chemicals [13–15]. Regrettably, this approach not only requires the utilization of toxic liquid chemicals but also confronts challenges associated with the generation of large amounts of waste acids and salts, along with tedious work-up procedures. Another approach toward OPCs is that the reaction of P_4_ with a strong base generates PH_3_, which is then functionalized by reactions with alkenes [14,15]. This method suffers from the wasteful utilization of phosphorus atoms, the inherent toxicity and the challenge in handling of PH_3_ gas (Fig. 1B).

In light of the urgent environmental concerns associated with conventional approaches, an alternative route directly converting P_4_ into OPCs has garnered both scientific significance and practical value. Remarkable progress has been made toward the formerly elusive goal of selectively transforming the P_4_ molecule in order to avoid the production of downstream chlorinated chemicals [16–19]. The pioneering efforts in this direction date back to the 1960s, employing specific organic molecules and simple organometallic reagents to activate P_4_ and yield certain OPCs [20]. Nevertheless, these methods encountered challenges of low yields and poor selectivity. In subsequent stages, metal-mediated P_4_ activation emerged as the predominant approach [21–23]. The allure of this methodology lies in its potential for achieving metal-catalyzed P_4_ transformation through the coordination and activation of P_4_ by a metal complex. While early transition metal (TM)-mediated P_4_ activation can lead to the formation of P‒H and P‒C bonds, the metal-catalyzed process remains a distant prospect. In the past 15 years, the introduction of highly active species, such as carbenes and their analogs, has enabled selective P_4_ activation [24].

Despite the significant progress in the field of P_4_ activation making OPCs or organometallic phosphorus compounds, it is still a highly challenging research area. In fact, it generally suffers from: (i) the high electrophilic reactivity of the P_4_ tetrahedron; (ii) the low selectivity for the P–P bond rupture after the first P–P bond cleavage; and (iii) the low conversion efficiency of the phosphorus atoms in P_4_ [25]. Consequently, the controllable and atom-efficient functionalization of P_4_ to construct directly OPCs or organometallic phosphorus compounds is highly desirable. Currently, there are over 30 research groups actively engaged in P_4_ activation and transformation internationally, mainly distributed in the United States [26–33], Germany [34–44], Russia [45–47], France [48,49], the Netherlands [50,51], the United Kingdom [52–58] and other countries [59–66]. China presently contributes over 70% to the global production of P_4_. However, the research of P_4_ activation and transformation in China lags behind. Our group first launched the study in this field in 2014, and the first paper entiteld “Direct Synthesis of Phospholyl Lithium from White Phosphorusˮ was published in 2016 [67]. Since then, we have reported a series of works on direct functionalization of P_4_ to constuct P‒C bonds [68–77]. In the past 5 years, 10 research groups in China have been involved in P_4_ activation and transformation [78–100]. Although some reviews have summarized the activation and transformation of P_4_ from different perspectives [16–25], there is no specific review summarizing progress in China. This timely review outlines the contributions of Chinese research teams in the field of P_4_ activation (Fig. 1C), and discusses the significance of P_4_ chemistry along with possible directions for future research. The objective of this review is to inspire increased involvement from researchers in advancing environmentally sustainable methods for P_4_ activation, fulfilling the desire to completely abandon the environmentally unfriendly industrial chlorination route in the future.

## CONSTRUCTION OF P–C BONDS

The construction of P‒C bonds from P_4_ is of great significance in both industry and academic research. However, due to the complex P–P bond breaking patterns, it is still challenging to construct P‒C bonds from P_4_ directly with good selectivity. Therefore, finding proper methods to deal with this challenge is necessary. During the past few decades, there has been some development in the construction of P–C bonds from P_4_ directly, including the reactions of organometallic reagents and organic molecules with P_4_.

### Reactions of P_4_ with organo-*di*-lithium reagents

**Figure 2.**
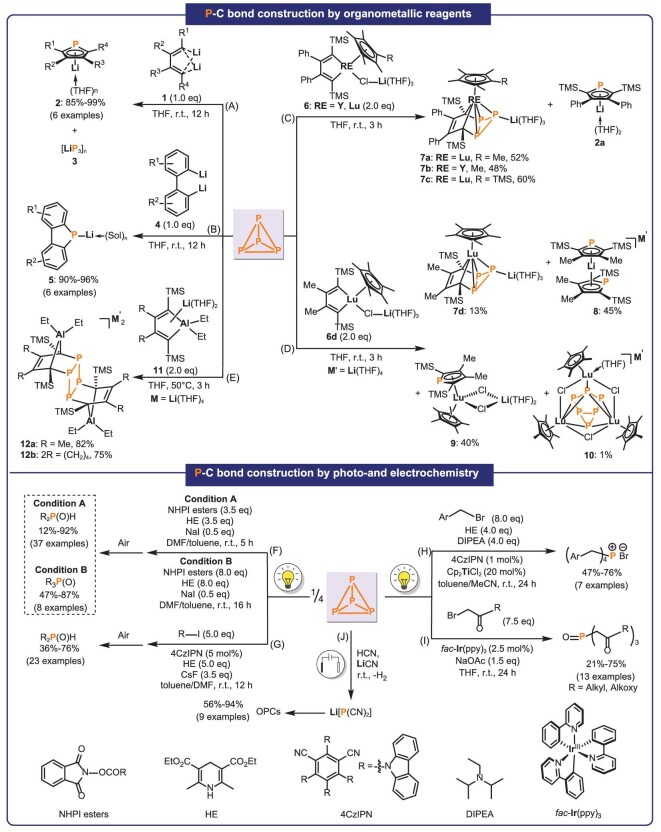
The direct construction of P–C bonds from P_4_. TMS, trimethylsilyl; NHPI, *N*-hydroxyphthalimide; HE, hantzsch ester; DIPEA, *N,N*-diisopropylethylamine.

In 2016, we reported for the first time the reaction of P_4_ with 1,4-dilithio-1,3-butadienes **1**, which quantitatively generated phospholyl lithiums **2** through the cooperative nucleophilic attack of two C_sp2_‒Li bonds on P_4_ (Fig. 2A) [67]. Di- or tetra-substituted phospholyl lithiums with diverse alkyl, aryl, or silyl groups can be prepared efficiently. Additionally, a novel cooperative insertion mechanism in the reaction of P_4_ with **1** involving the release of intermediate **3** was proposed and confirmed by density functional theory calculations. The understanding of this process will open a new door for the design of straightforward synthesis of OPCs from P_4_. Then, the aggregation states of phospholyl lithiums were systematically studied, revealing their potential to exist as monomers, dimers, and coordination polymers by changing the substituents and crystallization temperatures [68]. These investigations could be helpful when synthesizing other metal complexes supported by these phospholyl ligands. Furthermore, the [LiP_3_]_n_ moiety **3** was isolated and characterized as a mixture of phosphorus cluster anions, including Li_3_P_7_, Li_4_P_14_, Li_2_P_16_ and Li_4_P_26_ based on X-ray diffraction analysis and ^31^P{^1^H} COSY NMR analysis [69]. In 2022, the reaction of P_4_ with biphenyl dilithio reagents **4** was realized to produce a series of phosphafluorenyl lithiums **5** (Fig. 2B) [70]. The aggregation states of phosphafluorenyl lithiums were initially studied, and the obtained phosphafluorenyl lithiums were key synthetic intermediates for phosphafluorenes. In the same year, we further achieved the derivatization of phosphafluorenyl lithiums. When the one-pot reaction among P_4_, biphenyl dilithio reagents and ArCOCl or polyfluorobenzenes was carried out, the phosphafluorenyl-based acylphosphine oxides and triarylphosphines were obtained in a chlorine-free method [71]. The acylphosphine oxides and triarylphosphines could be used as radical photoinitiators and organophosphorus ligands.

### Reactions of P_4_ with rare-earth metallacyclopentadienes

Considering that metallacyclopentadienes can both work as double nucleophiles and dienes, we speculated that two M–C_sp2_ bonds could clip out a P_1_ fragment, forming a phospholyl anion via the aromatization driving force while the diene skeleton would trap the *cyclo*-P_3_ moiety to form an organosubstituted *cyclo*-P_3_ compound. Thus, we decided to investigate the reactions of rare-earth metallacyclopentadienes **6** with P_4_. Using this strategy, we synthesized the first series of rare-earth metal *cyclo*-P_3_ complexes **7** and phospholyl anion **2a** (Fig. 2C) [72]. In this process, the cleavage of three P‒P bonds and formation of four P‒C bonds for [3 + 1]-fragmentation were observed [73]. Complexes **7** represent the first *cyclo*-P_3_ complexes of rare-earth metals and also the first organo-substituted polyphosphides in the category of group 3 and f-block elements. The characterization of **7** suggests that dienes in **6** can trap the *cyclo*-P_3_ moiety and confirms the existence of released [LiP_3_] moiety in the reactions of P_4_ with 1,4-dilithio-1,3-butadienes **1**. To investigate the substituent effect of lutetacyclopentadienes, the reaction of Me, trimethylsilyl (TMS)-substituted lutetacyclopentadiene with P_4_ was carried out (Fig. 2D) [74]. The expected *cyclo*-P_3_ lutetium complex **7d** and the aggregated lithium phospholide **8** were obtained. Besides, the sandwich lutetium complex **9** was isolated as a new complex. Interestingly, an unexpected trinuclear rare-earth metal complex **10** with a *bicyclo*-P_6_^4−^ ligand was also observed in this reaction.

### Reactions of P_4_ with aluminacyclopentadienes

To investigate the importance of metal centers in metallacyclopentadienes for the direct functionalization of P_4_, the reactions of aluminacyclopentadienes **11** with P_4_ were carried out. Unexpectedly, the cyclotetraphosphanes **12** featuring four newly formed P–C bonds and a planar square *cyclo*-P_4_ ring was obtained selectively (Fig. 2E) [75]. These cyclotetraphosphanes represent an important class of organic polyphosphanes which are not easy to access by other methods.

### Photo- and electrochemical transformation of P_4_

Recent years have witnessed promising outcomes in the photo- and electrochemical transformation of P_4_. In 2022, the reactions of *N*-hydroxyphthalimide (NHPI) esters with P_4_ were developed by Tang *et al.* (Fig. 2F) [78]. This process, conducted without TMs or photocatalysts, resulted in a series of dialkyl and trialkyl phosphine oxides under blue light irradiation, with yields up to 92%. This synthetic method features the simple operation, broad substrate scope and high product selectivity. In 2023, another approach to construct dialkylphosphines was reported (Fig. 2G) [79]. Using organic-dye 4CzIPN as the photocatalyst, the unactivated alkyl iodides could react with P_4_ to generate dialkylphosphines in moderate to good yields. In the same year, we reported a method for the construction of tetrabenzylphosphonium bromide under blue light irridiation (Fig. 2H) [76]. Catalyzed by Cp_2_TiCl_2_ and 4CzIPN, the reactions between P_4_ and benzyl bromides produced a series of quaternary phosphonium salts, applicable in organic synthesis and pharmaceuticals. This marked the introduction of the metallaphotoredox catalysis strategy for the first time in the field of P_4_ activation. Additionally, we also reported the synthesis of phosphoryltriacetates in 2023 (Fig. 2I) [77]. Using *fac*-Ir(ppy)_3_ as the photocatalyst and blue LEDs (456 nm) as the light source, P_4_ can react with α-bromo esters to generate phosphoryltriacetates in the one-step reaction with moderate to good yields.

Moreover, electrochemical transformation of P_4_ to OPCs was reported by Liu *et al.* in 2022 (Fig. 2J) [80]. P_4_ was first electro-oxidized into [P(CN)_2_]^−^, which was subsequently used to synthesize the useful OPCs, such as phospholides and cyclophosphanes. Notably, this method can accomplish the activation of P_4_ on a gram scale.

## CONSTRUCTION OF P–S/N/O BONDS

Heteroatom-containing OPCs, such as phosphorothioates (C–S–P bonds), phosphoramidates (C–N–P bonds), or phosphates (C–O–P), have important applications in the fields of medicinal chemistry, ligands, organocatalysis and agrochemistry [101–103]. The synthetic methods of these compounds mainly involve two routes: (i) the substitution reactions of PCl_3_, and (ii) the classical coupling reactions, free radical reactions or substitution reactions of specific phosphorus reagents, such as (RO)_2_P(O)H, P(OR)_3_ or R_2_P(O)H, etc. [104–106]. However, it is noteworthy that most phosphorus-containing reagents used in the currently developed methods for the synthesis of OPCs must also be pre-prepared from PCl_3._

### Construction of P–S/N bonds

**Figure 3.**
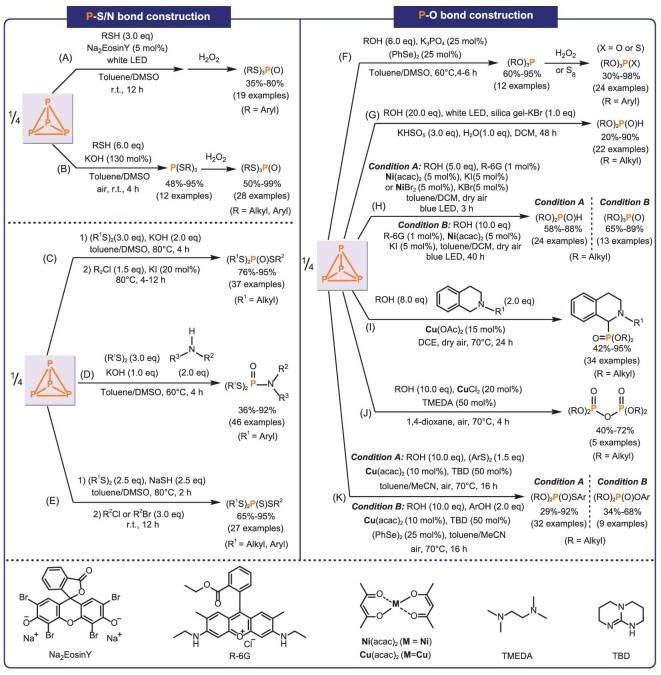
The direct construction of P–S/N/O bonds from P_4_. TMEDA, *N,N,N*',*N*'-tetramethylethylenediamine; TBD, 1,5,7-triazabicyclo[4.4.0]dec-5-ene.

In 2019, Tang *et al.* first reported a photocatalytic reaction of P_4_ for the construction of P–S bonds (Fig. 3A) [81]. The phosphorotrithioates were synthesized by direct reaction of P_4_ and thiophenol using Na_2_EosinY as a photocatalyst. However, the substrate scope of this method was limited, which was only suitable for thiophenol, but mercaptan could not give the corresponding products. Subsequently, they reported the synthesis of phosphorotrithioates under mild conditions with KOH or K_2_CO_3_ as base and air as oxidant (Fig. 3B) [82]. Both thiophenol and mercaptan could be tolerated, and the yields were almost quantitative.

The above methods have successfully realized the synthesis of P(SR)_3_ and P(O)(SR)_3_ with the same substituents, but the synthetic method of mixed phosphorotrithioates (R^1^S)_2_P(O)SR^2^ was still worth exploring. In 2020, they reported the synthesis of mixed phosphorotrithioates (R^1^S)_2_P(O)SR^2^ by a one-pot, two-step procedure from P_4_, disulfide and alkyl halides with KOH as the base (Fig. 3C) [83]. The key intermediate in this process is (R^1^S)_2_P(S)OK, which was generated by the attack of RSK on *S,S,S*-trialkyl phosphorotrithioates and subsequent C–S bond cleavage (Michaelis–Arbuzov-like dealkylation reaction). In addition, they considered that penta-coordinate ionic species may be formed during the reaction, which may exchange with KOH to form the P=O bond. To explore this feasibility, amines were introduced in this reaction, which may exchange with the –SPh group to form a P–N bond. Based on this, they reported the efficient four-component synthesis of phosphoramidodithioates from P_4_, disulfides, amines and KOH (Fig. 3D) [84]. KOH serves both as the base in the reaction and as the source of oxygen atoms in the products. After that, they further synthesized (R^1^S)_2_P(S)SR^2^ with NaSH instead of KOH under reaction conditions similar to Fig. 3C. The NaSH provided the sulfur atoms in the products (Fig. 3E) [85].

### Construction of P–O bonds

In 2021, Tang *et al.* envisioned that the catalytic activation of P_4_ with (RSe)_2_ might produce P(SeR)_3_ species, which can undergo further nucleophilic substitution with ArOH, eventually leading to P(OAr)_3_ products. Based on that, using (PhSe)_2_ as the catalyst and dimethyl sulfoxide (DMSO) as both the solvent and oxidant, they realized the synthesis of triaryl phosphites and triaryl phosphates from P_4_ and aryl phenol with almost quantitative yield (Fig. 3F) [86]. The triaryl phosphites could be oxidized with H_2_O_2_ or S_8_ to afford the corresponding oxidation products triaryl phosphates and triaryl thiophosphates. While this work provided a straightforward synthesis of triaryl phosphites, the substrate scope is limited to phenol compounds, and alcohols are not applicable. Dialkylphosphites are widely used as basic starting materials for the synthesis of complex phosphate-based organic compounds. Tang *et al.* reported the direct synthesis of dialkylphosphites from P_4_ and alcohol. The reaction was mediated by KBr with KHSO_5_ (oxone) as the oxidant (Fig. 3G) [87]. In this process, oxone and KBr were employed to *in situ* produce PBr_3_ intermediate, thus replacing the traditional PCl_3_ route.

To avoid the consumption of a large amount of oxidants, Tang *et al.* developed a waste-free, environmentally friendly method for the synthesis of dialkylphosphites from P_4_ and alcohols. This approach utilized a combination of photoredox catalyst, nickel catalyst and halide anion under visible light (Condition A; Fig. 3H) [88]. The trialkylphosphate could be obtained by increasing the amount of alcohol and prolonging the reaction time (Condition B; Fig. 3H). This photocatalytic method proved to be effective for various alcohols. Furthermore, it demonstrated suitability for the synthesis of phosphorotrithioates.

*α*-Aminophosphonates find broad applications in fungicides and enzyme inhibitors. In 2023, Tang *et al.* reported a Cu-catalyzed three-component reaction among tetrahydroisoquinolines, P_4_, and alcohols to synthesize *α*-aminophosphonate with air as a safe oxidant (Fig. 3I) [89]. Furthermore, the method was also suitable for the selective construction of P–O–P compounds (Fig. 3J) [89]. Recently, Tang *et al.* described a novel and high-yielding method for the synthesis of various phosphorothioates from P_4_, disulfides and alcohols in one step (Condition A; Fig. 3K) [90]. They hypothesized the formation of phoroselenoate derivatives when replacing diaryl disulfides with diphenyl diselenides. Subsequent nucleophilic substitution with ArOH would result in mixed alkyl/aryl phosphates. Based on this hypothesis, they successfully synthesized the corresponding mixed phosphates using diphenyl diselenides as catalyst and phenol as nucleophile (Condition B; Fig. 3K). This method achieves the simple and efficient synthesis of phosphorothioates with diverse structures.

## CONSTRUCTION OF P–M/E BONDS

In the past decade, the activation and transformation of P_4_ by main group element (E) and metal (M) complexes has attracted intense attention and been subjected to extensive study in the Chinese chemistry community. This is due to their potential utility in the synthesis of OPCs and metal phosphide materials. Various P_n_-containing compounds with different nuclearities and geometries have been prepared. It not only provides an environmentally friendly and straightforward route to synthesize high–value-added OPCs without the utilization of poisonous and corrosive chlorine gas but also leads to the production of various metal phosphides with marvelous structures and reactivities [18–25]. It should be noted that the organometallic complexes-mediated P_4_ activation always involves complex processes, including the cleavage and formation of several bonds. The resulting metal phosphide complexes are intimately related to the type of metal complexes and the coordination environment. Additionally, there is relatively little research on the mechanisms of these processes, highlighting an area where more investigation is needed in the future.

### Construction of P–E bonds

**Figure 4.**
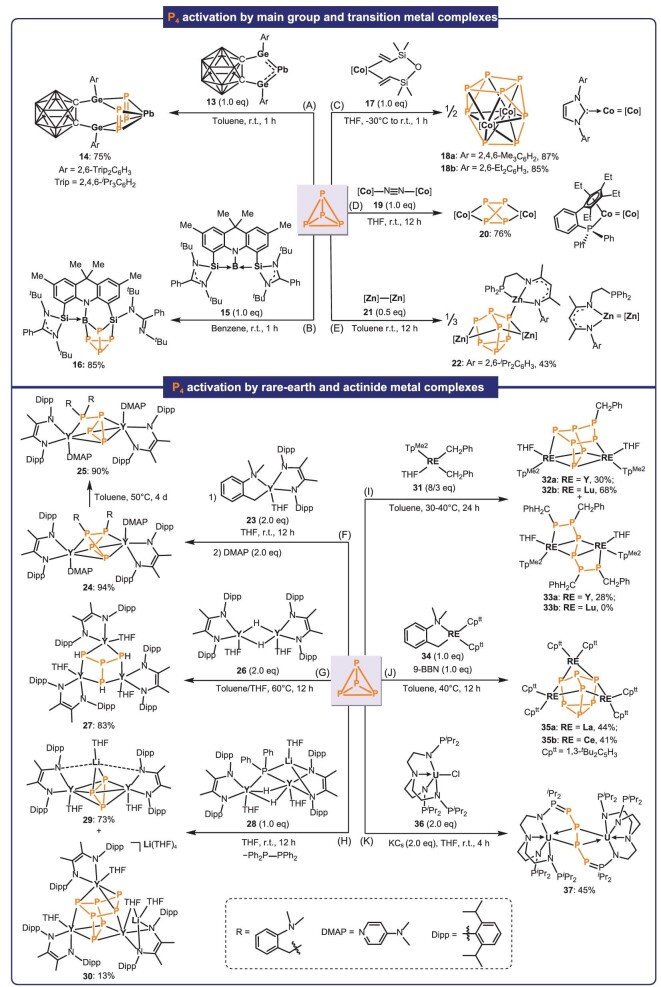
Activation and functionalization of P_4_ with metal complexes. RE, rare-earth.

The activation of P_4_ by main group elements is an established field of chemistry [20]. In 2023, Mo *et al.* reported the synthesis of an elusive homoleptic diphosphene lead complex **14** through controllable degradation of P_4_ by zero-valent lead complex **13** at ambient temperature (Fig. 4A) [91]. Complex **14** possesses significant π bonding between the Pb atom and diphosphene ligands, with Pb→P_2_ π-backbonding and P_2_→Pb σ-donation. The utility of diphosphene as a π-electron donor to stabilize low-valence lead complexes provides a new strategy to develop π-complexes of main group elements.

In the same year, they successfully synthesized a geometrically constrained borylene **15** with the help of a rigid pincer bis(silylene)amido ligand. The reaction of **15** with P_4_ afforded the product **16** in 85% yield; **16** possesses a BSiP_4_ cage, which is formed by the insertion of the P_4_ molecule into the Si–B bond (Fig. 4B) [92].

### Construction of P–TM bonds

The TM-mediated activation of P_4_ has given rise to a plethora of fascinating complexes bearing versatile P_n_ units. The synthesis of these P_n_-containing compounds from P_4_ necessitates the cleavage of one or more P–P bonds, and possibly the formation of new P–P bonds [19].

In 2022, Deng *et al.* reported P_4_ activation with three-coordinate *N*-heterocyclic carbene (NHC)-cobalt(0)-alkene complexes **17**. This reaction selectively produces the large polyphosphorus cobalt clusters **18** bearing P_8_ ligands in high yields. These Co_2_P_8_ clusters feature short Co–Co bonds (2.39 Å), and P_8_ ligands exhibit signet-ring type geometry that is unprecedented in synthetic P_8_ complexes (Fig. 4C) [93]. A series of cage functionalization reactions were conducted and indicated the amphiphilicity of the P_8_ ligands in **18** toward electrophiles and nucleophiles. A possible mechanism for the formation of **18** was proposed. P_4_ reacts with one molecule of **17** to generate *cyclo*-P_4_ intermediates, which can interact with a second molecule of **17** to afford the *chain*-P_4_ species. The *chain*-P_4_ species feature two coordinatively unsaturated cobalt centers and can interact with P_4_ to produce the final products. As an alternative route, the direct dimerization of *cyclo*-P_4_ intermediates could also give the P_8_ complexes. In the same year, Xi *et al.* synthesized and structurally characterized the dinuclear cobalt dinitrogen complex **19** bearing cyclopentadienyl-phosphine ligands. The two cobalt centers of **19** could undergo oxidative addition of two individual P–P bonds in the P_4_ moiety resulting in the formation of diamagnetic complex **20** with a *cyclo*-P_4_ moiety (Fig. 4D) [94]. Very recently, Xu *et al.* reported the first example of P_4_ activation by a group 12 metal-centered complex. They found that the Zn(I)−Zn(I) bonded compound **21** could serve as a two-electron reducing reagent to selectively reduce a number of small molecules, including P_4_. The reaction of **21** with P_4_ gave a trinuclear zinc complex **22**, which contains a *zintl*-P_7_ ligand (Fig. 4E) [95].

### Construction of P–RE bonds

A combination of coordinative unsaturation and inherent Lewis acidity of rare-earth (RE) metals, and the strong nucleophilicity of RE−C bonds, endows rare-earth organometallics with a rich reaction chemistry toward P_4_. Rare-earth metal-mediated P_4_ activation can lead to the formation of P‒H and P‒C bonds. Hence, this approach has attracted significant interest [27].

In 2019, Zhou *et al.* synthesized for the first time a rare-earth organonometallic *cyclo*-P_4_ complex **24** by direct functionalization of P_4_ using a rare-earth metal alkyl precursor **23**. Heating **24** at 50°C in toluene for 4 days afforded the R_2_P-substituted *cyclo*-P_3_ complex **25** in 90% yield through alkyl migration. This transformation provides a new insight into the stepwise degradation of P_4_ using metal complexes (Fig. 4F) [96]. In 2023, they reported the reaction of yttrium hydride **26** with P_4_, which results in the formation of a trinuclear yttrium complex **27** bearing a unique pyramid-like P(PH)_3_ moiety (Fig. 4G). In contrast, the intramolecular cooperative yttrium hydride/LiPPh_2_-mediated P_4_ activation results in the production of two multinuclear heterometal polyphosphorus complexes, one with *cyclo*-P_3_ fragment (**29**) and the other with norborane-P_7_ fragment (**30**) (Fig. 4H). These findings showcased the synergistic effect of rare-earth hydride and LiPPh_2_, introducing a new mode of P_4_ activation [97].

Very recently, Zhou *et al.* successfully synthesized two novel rare-earth polyphosphides through the direct P_4_ activation with rare-earth metal dialkyl complexes **31**. Treatment of the yttrium dialkyl complex **31a** with P_4_ in toluene at ambient temperature resulted in the formation of two yttrium polyphosphorus complexes: norbornene-BnP_7_ complex **32a** or chain-Bn_4_P_6_ complex **33a**, respectively. Notably, the analogous reaction of **31b** with P_4_ in toluene at 40°C only gave **32b**. These results illustrated the important influence of the metal centers on the reactivity of the organometallic compounds (Fig. 4I) [98].

In 2022, Ren *et al.* investigated the reduction of P_4_ using *in situ* generated lanthanum and cerium hydrides. Treatment of the lanthanocene or cerocene alkyl complexes **34** with P_4_ followed by the addition of 9-BBN afforded the trinuclear lanthanide complexes **35a, b** with a *μ*-bridging P_7_^3−^ ligand (Fig. 4J) [99].

### Construction of P–An bonds

The study of small molecule activation by actinide (An) elements still lags far behind that of main-group elements and TMs. Reports on the P_4_ activation by uranium species are rare. In 2021, Zhu *et al.* reported the formation of a uranium polyphosphide **37** with an *E*-type P_4_ chain by treating the uranium chloride **36** with P_4_ and KC_8_ in tetrahydrofuran (THF). Computational studies showed that the U(III)–P(III) synergistic effect allows a direct six-electron reduction of P_4_ (Fig. 4K) [100]. This study further demonstrates the ability of the synergistic strategy between metal centers and ligands to activate P−P bonds, which may inspire the design of new systems for P_4_ activation.

## CONCLUSION AND OUTLOOK

The direct conversion of P_4_ into P-containing compounds holds paramount significance both in terms of fundamental understanding and practical applications. Over the past six decades, substantial progress has been made in directly forming OPCs from P_4_. Early efforts faced challenges of poor selectivity and low yields due to the unique tetrahedral structure and high reactivity of P_4_. The prospect of transition metal-catalyzed P_4_ transformations remain unresolved challenges due to the intricate nature of the reactions between low-valence TMs and P_4_. By the delicate substrate and pattern design, some highly selective conversions of P_4_ to OPCs have been developed, yet these reactions are stoichiometric. In recent years, catalytic reactions producing mono-phosphorus compounds from readily available substrates and P_4_ have made notable progress through photochemical and electrochemical approaches, garnering widespread attention. However, these intriguing reactions still exhibit some drawbacks, such as the need for substantial additives or substrates, difficulties in scaling up, and limited applications of the products. Therefore, the development of more efficient and environmentally friendly systems is imperative. Additionally, exploring new reaction types is essential. In this context, we propose that the following fields can be considered in the future.

### Mechanistic studies on P_4_ degradation

While the conversion of P_4_ to mono-phosphorus compounds represents a promising direction, the progress in understanding the complex and unclear mechanism of P_4_ degradation is limited. In-depth mechanistic studies through the combination of *in situ* characterization techniques and computational chemistry would greatly aid in the development of direct synthesis of mono-phosphorus compounds from P_4_.

### New reaction systems

Most current catalytic processes for OPCs from P_4_ rely on radical systems. This preference arises from the fact that, compared to other reactive species, radicals are electrically neutral, allowing a single radical to react with P_4_ to produce neutral organophosphorus product without the consideration of charge conservation. This process always leads to the singularity of P-atom connecting groups. Furthermore, this strategy also suffers from the poor reactivity caused by the low efficiency of free radical formation. Exploring P_4_ transformations induced by other *in situ* generated active species, such as carbenes, frustrated radical pairs, etc., is an intriguing research topic.

### Electrochemistry

The transformation of P_4_ into OPCs involves changes in the oxidation state of phosphorus atoms. In terms of redox chemistry, which is frequently encountered when forging new bonds, it is difficult to conceive of a more economical way to add or remove electrons than electrochemistry. Therefore, the development of electrochemical methods for P_4_ aligns with the requirements of sustainability and economics. Meanwhile, electrochemistry serves as a potent tool for generating active species, holding great promise in the realm of P_4_ functionalization. One straightforward concept is the reduction of P_4_ to [P]^3−^ species at the cathode, which can then react with electrophiles in the system to produce the final organic compounds.

### *In situ* transformation via P-protonation, P-sulfenylation or P-chlorination intermediates

Inspired by the work of the conversion of P_4_ into organophosphate compounds via the P-sulfenylation and P-selenylation intermediates, this strategy can be further expanded. Such a strategy can capitalize on established conversion methodologies, streamlining synthetic procedures, while simultaneously preventing the formation of toxic by-products and waste acids or salts.

### Merging C–H activation with P_4_ transformation

Among the reported catalytic transformations of P_4_ to OPCs, the prerequisite for substrates to possess leaving groups often results in diminished atom economy. While C–H bond activation has witnessed significant advancements in modern synthetic chemistry, its amalgamation with P_4_ activation remains a challenging and largely unexplored area. The C–H activation-based transformations of P_4_ will streamline the synthetic routes to OPCs, significantly enhancing atom economy and synthetic efficiency. For instance, the conventional synthesis of triphenylphosphine involves the use of sodium and chlorobenzene. Direct synthesis of triphenylphosphine from benzene and P_4_ would yield substantial economic and environmental benefits.

### Machine learning for P_4_ activation

The application of machine learning in the fields of chemistry and materials science is rapidly expanding, bringing unprecedented innovation and progress to these disciplines. In the realm of P_4_ activation, machine learning can be employed for the design of reaction pathways, enhancement of catalyst performance, optimization of reaction conditions, etc., thereby accelerating the discovery of environmentally friendly and efficient methods for P_4_ transformation.

### Homogeneous–heterogeneous synergy strategy for P_4_ activation

Heterogeneous chemical reactions excel at facile cleavage of inert bonds, whereas homogeneous chemical reactions are adept at synthesizing fine chemicals. In recent years, a strategy combining homogeneous and heterogeneous approaches has been successfully applied to the synthesis of nitrogen-containing compounds from N_2_ [107,108]. If this new strategy could be implemented in the field of P_4_ activation, it would offer fresh opportunities for the direct synthesis of high-value OPCs from P_4_ and simple organic molecules.
